# A Noble Institution

**Published:** 1888-10

**Authors:** 


A NOBLE INSTITUTION.—Under this caption the *Atlanta Evening Journal* makes the following laudatory remarks in regard to the Ivy Street Hospital, which is connected with that excellent medical institution known as the Southern Medical College: "We are confident that all good citizens of Atlanta will rejoice to learn that this hospital is now being placed on so sure and excellent a footing, rendering it just the hospital needed for the treatment of all classes of diseases requiring scientific practitioners of the healing art.

Since the creation of this institution it has, in a measure, been crippled by its humane policy, the big heart of its president, Dr. T. S. Powell, having prompted him to make it a home for the poor and homeless sick. When the city committed its paupers to the care of the Ivy Street Hospital, he received them to the injury of the institution, willing not only to afford homes and sustenance and medical treatment to the afflicted poor. both white and colored, but to save the city great trouble and expense in giving attention to this unfortunate class of citizens.

The city authorities have found other and still cheaper methods of providing for the sick paupers in our midst, suspended the relations existing between them and Ivy street hospital, and in doing this have set the institution free from an injurious policy, and given it an impetus in the way of improvements and progress which will cause its friends to rejoice. Now it will be run as a private hospital, and be under the management of a business committee, who are now preparing to refit and refurnish it in every department, and rendering it a suitable refuge for all who may seek its advantages, however wealthy and refined.

The medical faculty of the Southern Medical College have agreed to divide the work and relieve Dr. Powell as much as possible, who has hitherto borne, at great labor and sacrifice, the exclusive management and control of this institution. These gentlemen have in a recent official meeting recognized in most appreciative and complimentary terms, the work of this noble man, and have expressed the determination to divide the duties and labors he has so long discharged with a wise discretion and unquestionable fidelity.

They well knew when they presented him the beautiful medal of virgin gold with the motto "*Vertus in Arduis*" (strong in difficulties), the true character of their president, for he has in every emergency plainly manifested his great ability to grapple with and overthrow unjust opposition, that all who know him have learned that his energies and his resolution know not how to yield when his cause or his purpose is right. All of the friends of Ivy street hospital and the Southern Medical College may rest assured that he will never succumb to the enemies of this institution, but that he will continue to improve it in every detail and advance its interest until it is the pride not only of all good citizens of Atlanta, but of the entire South.

The hospital will be furnished in splendid style and equipped with every convenience, so that the afflicted may find it a home equal to any. The ladies who from the beginning have given Dr. Powell their aid in establishing this institution, will assist, no doubt, the faculty in bringing it to the very highest degree of excellence.

